# A Compact Double-Folded Substrate Integrated Waveguide Re-Entrant Cavity for Highly Sensitive Humidity Sensing

**DOI:** 10.3390/s19153308

**Published:** 2019-07-27

**Authors:** Zhihua Wei, Jie Huang, Jing Li, Junshan Li, Xuyang Liu, Xingsheng Ni

**Affiliations:** College of Engineering and Technology, Southwest University, Chongqing 400715, China

**Keywords:** microwave, humidity sensor, folded SIW, re-entrant cavity resonator

## Abstract

In this study, an ultra-compact humidity sensor based on a double-folded substrate integrated waveguide (SIW) re-entrant cavity was proposed and analyzed. By folding a circular re-entrant cavity twice along its two orthogonally symmetric planes, the designed structure achieved a remarkable size reduction (up to 85.9%) in comparison with a conventional TM_010_-mode circular SIW cavity. The operating principle of the humidity sensor is based on the resonant method, in other words, it utilizes the resonant properties of the sensor as signatures to detect the humidity condition of the ambient environment. To this end, a mathematical model quantitatively relating the resonant frequency of the sensor and the relative humidity (RH) level was established according to the cavity perturbation theory. The sensing performance of the sensor was experimentally validated in a RH range of 30%–80% by using a humidity chamber. The measured absolute sensitivity of the sensor was calculated to be 135.6 kHz/%RH, and the corresponding normalized sensitivity was 0.00627%/%RH. It was demonstrated that our proposed sensor not only has the merits of compact size and high sensitivity, but also benefits from a high Q-factor and ease of fabrication and integration. These advantages make it an excellent candidate for humidity sensing applications in various fields such as the agricultural, pharmaceutical, and food industries.

## 1. Introduction

Humidity is one of the most important indexes for evaluating environmental quality, and it is crucial to monitor and control humidity accurately in many scenarios. For instance, the relative humidity (RH) level in greenhouses is a key factor that affects the growth and disease incidence of crops [1]. For food storage, the RH needs to be controlled at a proper level so as to prevent the putridity of foods [2]. Other fields such as material processing, industrial manufacturing, as well as electrical equipment operating also have rigorous requirements on the RH of the environment [3,4]. Within this context, developing humidity sensors with high performance has aroused considerable enthusiasm and become a subject of intensive research.

Traditional humidity sensors involve various types including resistive [5,6,7], capacitive [8,9,10], quartz crystal microbalance (QCM) [11,12], piezoelectric [13], and so on. Most of these sensors, however, operate at very low frequencies (quasi-direct current (DC)).This makes them difficult to be utilized directly in modern radio frequency (RF) sensing and detection systems. Fortunately, the passive microwave sensor has been recently exploited and developed as a promising alternative for RH detection. The passive microwave sensor can not only be conveniently applied to RF sensing systems, but also offers the merits of low cost, high design flexibility, and freedom from a power supply. Thus far, a number of passive microwave RH sensing schemes have been reported [14,15,16,17,18,19,20,21,22,23,24,25,26]. One branch of this research concentrates on the synthesis of novel sensing materials and their combinations with diverse microwave components; it is dedicated to improving the sensing performance of sensors (e.g., sensitivity, resolution, etc.). A variety of sensing materials such as polyvinyl-alcohol (PVA) [14], poly (3,4-ethylenedioxythiophene): poly (styrene-sulfonic acid) (PEDOT:PSS) [15], and silicon nanowires [16] have been investigated. Despite the contribution to sensing performance improvement, the usage of sensing materials inevitably increases the production cost and fabrication complexity of the sensors. Meanwhile, the effective RH sensing ranges of these sensors may also be limited by the sensing materials, which hinders their practical applications to some extent. Therefore, remarkable efforts have been devoted to develop RH sensors using novel, passive microwave structures without sensing materials [22,23,24,25,26].

Among various topologies, substrate integrated waveguide (SIW) has gained increasing attention due to its low cost, low profile, high power capacity, and easy integration with other planar circuits. An SIW cavity available for RH sensing was first presented by Matbouly et al. [22]. Thereafter, a modified air-filled SIW cavity was proposed with the aim of improving sensitivity [23]. Through fully replacing the middle substrate of the cavity with moist air, a sensitivity up to 1.21 MHz/%RH (with an operating frequency of 7.63 GHz) was successfully realized. Nevertheless, the overall sizes of these structures are too large, making them unsuitable for compact integrated systems. As an attempt to address this issue, Jones et al. proposed a ridged quarter-mode SIW (QMSIW) humidity sensor [24]. By introducing the ridged quarter-mode technique, it achieved a size reduction of 86.2% compared to the sensor reported in [22]. However, this design suffers from a poor sensitivity (only 30.73 kHz/%RH at 6.9 GHz), and its loaded Q-factor is also relatively low due to the open boundaries inherent with quarter-mode structures. From these reported works, one can see that the realization of SIW humidity sensors simultaneously possessing high sensing performance and miniaturized size still remains a challenge.

Herein, we propose a novel double-folded SIW (DFSIW) re-entrant cavity and demonstrate its ability for highly sensitive humidity sensing both numerically and experimentally. The overall size of the cavity is dramatically miniaturized by combining the folding technology with re-entrant configuration. Numerical analyses show that it only occupies 14.1% of the overall size of conventional TM_010_-mode SIW circular cavities. The high sensitivity property of the sensor is achieved by introducing sensitive regions inside the cavity where the electric field is highly concentrated. When moist air enters the sensitive regions, it causes a strong perturbation to the electric field and results in a large resonant frequency shift of the cavity. This structural design offers an effective solution for balancing the sensing performance and physical dimensions of a passive microwave humidity sensor, which is of great significance for promoting the development and application of microwave humidity sensing devices.

## 2. Sensing Mechanism and Sensor Design

### 2.1. Sensing Mechanism

The working principle of the designed DFSIW re-entrant cavity humidity sensor is based on the cavity perturbation method. The moist air can be regarded as a dielectric medium, whose dielectric constant is related to *RH* (unit: %) as follows [3,22]:

(1) $$\varepsilon_{r}(\mathrm{RH})\;=\;1\;+\;\frac{211}{T}\left(P\;+\;48\frac{P_{s}}{T}\mathrm{RH}\right)10^{-6},$$

where T is the absolute temperature (unit: K). P and $P_{s}$ represent the pressures of moist air and saturated water vapor (unit: mmHg), respectively.

When the cavity is exposed to a humid environment, the moist air spreads into the sensitive region of the cavity, and then causes perturbation to the induced electric field of the cavity, finally leading to a remarkable change in the resonant frequency. According to the cavity-material perturbation theory [27,28], the fractional frequency shift can be approximately computed as:

(2) $$\frac{f_{r}-f_{0}}{f_{0}}\approx -\frac{\int_{V_{0}}^{\;}\left(\Delta \varepsilon E\cdot E_{0}^{*}+\Delta \mu H\cdot H_{0}^{*}\right)dv}{\int_{V_{0}}^{\;}\left(\varepsilon {\left|E_{0}\right|}^{2}+\mu {\left|H_{0}\right|}^{2}\right)dv},$$

where $f_{r}$ and $f_{0}$ represent the resonant frequency of the perturbed and unperturbed cavities, respectively. E(H) and $E_{0}$($H_{0}$) are the induced electric field (magnetic field) of the cavity with and without perturbation, respectively, while $E_{0}^{*}$ and $H_{0}^{*}$ are the complex conjugates of $E_{0}$ and$\;H_{0}$, respectively. $V_{0}$ represents the volume of the cavity. Δε and Δμ denote the variation in permittivity and permeability introduced by the air sample, respectively. As aforementioned, the moist air is considered as a dielectric medium, so the variation in permeability should be equal to zero (i.e., ∆*μ* = 0). Then, by using a quasi-static approximation of the field internal to the air sample [29], Equation (2) can be further simplified to

(3) $$\frac{f_{r}-f_{0}}{f_{0}}\approx -\frac{\int_{V_{s}}^{\;}\Delta \varepsilon E_{int}\cdot E_{0}^{*}dv}{2\int_{V_{0}}^{\;}\varepsilon {\left|E_{0}\right|}^{2}dv},$$

where $\;E_{int}$ is the electric field internal to the moist air at the sensitive region and $V_{s}$ is the effective volume occupied by the sample. The internal electric field $E_{int}$ is formed by the polarization effect of the air sample under the excitation of $\;E_{0}$, and it is mainly determined by the shape of the sample and the relative orientation between the sample and$\;E_{0}$. As for the designed sensor in this work, the shape of the air sample can be modeled as a thin circular sector slab with $E_{0}$ normal to it. For such a configuration, the $E_{int}$ associating with $E_{0}$ is given as [29]

(4) $$E_{int}=\frac{1}{\varepsilon_{r}\left(\mathrm{RH}\right)}E_{0},$$

Substituting Equation (4) into Equation (3), we finally obtain

(5) $$\frac{f_{r}-f_{0}}{f_{0}}\approx \;-\frac{1}{2}A\times \frac{\varepsilon_{r}(\mathrm{RH})\;-\;1}{\varepsilon_{r}\left(\mathrm{RH}\right)},$$

where *A* = $\frac{\int_{V_{s}}^{\;}{\left|E_{0}\right|}^{2}dv}{\int_{V_{0}}^{\;}{\left|E_{0}\right|}^{2}dv}$, and it is mainly affected by the volume ratio of the air sample to the cavity. Given that $V_{s}$ is always smaller than $\;V_{0}$, the value of *A* should be in the range of 0 ≤ *A* < 1. Once the geometrical parameters of the cavity are fixed, *A* would be kept as a constant. Its value can be determined accurately using a calibration method. The above theoretical model shows that the fractional frequency shift of the sensor is a function of the RH level. That is, the RH condition of the ambient air can be easily retrieved by measuring the corresponding resonant frequency variation of the sensor.

### 2.2. DFSIW Re-Entrant Cavity Design

**Figure 1:**
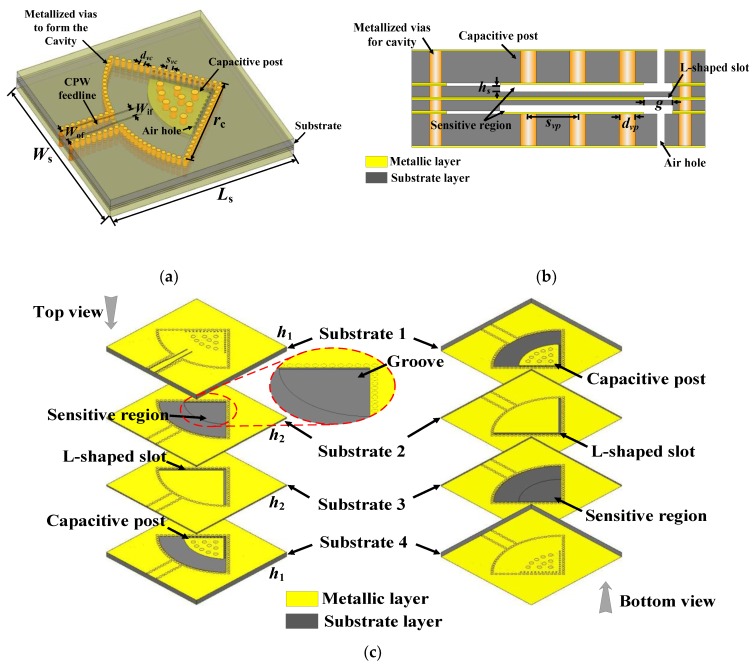
Diagram of the proposed double-folded substrate integrated waveguide (DFSIW) re-entrant cavity: (**a**) full view; (**b**) cutaway view (without feedline); (**c**) exploded view.

Figure 1 illustrates the geometrical structure of the proposed DFSIW re-entrant cavity humidity sensor, which is generated by folding a conventional circular re-entrant cavity twice along its two orthogonally symmetric planes. The structure consists of four dielectric substrate layers sandwiched between multiple metallic layers. The dielectric substrate used was F4B-2 with a dielectric constant of 2.65 and a loss tangent of 0.001, and the metallic layer was a copper film with a thickness of 35 µm. An L-shaped slot was etched on the bottom metallic layer of substrate 2 and the top metallic layer of substrate 3. This slot is the key element for realizing folded structures [30,31,32]. Its main function is to create fictitious magnetic walls that enable the propagation of electromagnetic waves from the upper substrates to the lower ones. In this manner, the desired electromagnetic field distribution in a folded form can be obtained. By choosing an appropriate value of the slot width, the folded cavity is able to achieve the similar resonant properties to its unfolded counterpart [33]. The sensitive regions for humidity detection were introduced in substrates 2 and 3, with the same area as the capacitive post. To allow the entrance of moist air into the cavity, two orthogonal rows of air holes were drilled on substrates 1 and 4. The ambient sidewalls of the cavity were created through a number of metallized vias that connect all the metallic layers. Note that the diameter of the via (*d*_vc_) and the spacing between two adjacent vias (*s*_vc_) should obey the constraint of $\frac{s_{\mathrm{vc}}}{2}\leq d_{\mathrm{vc}}<\frac{\lambda_{g}}{5}$, with $\lambda_{g}$ being the guided wavelength. This is to ensure that the radiation loss between adjacent vias can be neglected. Specifically, *d*_vc_ = 1 mm and *s*_vc_ = 1.4 mm were used in this design. The excitation of the cavity was accomplished using a tapered coplanar waveguide (CPW) feedline with a characteristic impedance of 50 Ω. The geometrical parameters of the final optimized cavity are listed in Table 1.

**Figure 2:**
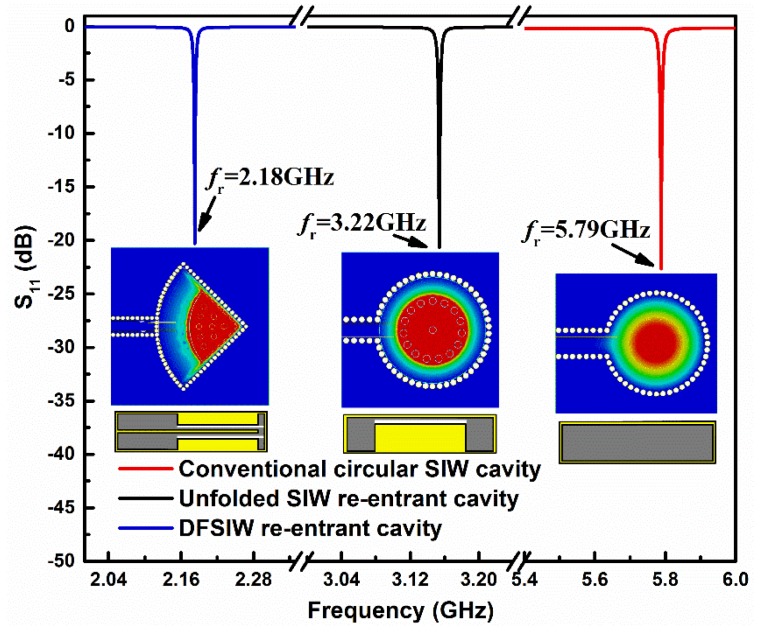
Simulated reflection coefficients S_11_ of a conventional circular SIW cavity, an unfolded SIW re-entrant cavity, and the proposed DFSIW re-entrant cavity. Note that the resonant cavity areas of these three cavities were kept identical for a better comparison. The insets are the corresponding electric distributions of these three cavities at resonances.

To quantitatively demonstrate the ability of the designed structure for size miniaturization, the resonant properties of three kinds of cavities (conventional circular SIW cavity, unfolded SIW re-entrant cavity, our proposed DFSIW re-entrant cavity) were investigated using the Finite Difference Time Domain (FDTD) method. Figure 2 shows the simulated reflection coefficients S_11_ of these three cavities, as well as their electric field distributions at resonances. For better comparisons, the resonant cavity areas and heights of these three cavities were set to be identical (158.76  π  mm^2^ ×  5 mm). From Figure 2, the circular SIW cavity operating in the TM_010_ mode resonated at 5.79 GHz, while the re-entrant cavity exhibited a lower resonant frequency of 3.22 GHz. This frequency shift, corresponding to a relative size reduction of 70.1%, is mainly attributed to the large equivalent capacitance introduced by the capacitive post of the re-entrant cavity [34]. Taking a further step, the re-entrant cavity was folded twice along its two orthogonally symmetric planes, forming the so-called DFSIW re-entrant cavity. Due to this folding operation, the resonant frequency of the cavity was further decreased to 2.18 GHz, which means that a further miniaturization of 54.2% was realized. It is emphasized once again that the resonant cavity area of the DFSIW re-entrant cavity was scaled up to 158.76 π mm^2^ to keep it identical with the cavity area of the unfolded re-entrant cavity, and the size miniaturization considered here is for the electrical size (i.e., relative size with respect to the resonant frequency). If its cavity area was not scaled, the DFSIW re-entrant cavity would have the same resonant frequency with the unfolded re-entrant cavity. This can also be demonstrated by the electric field distributions of the cavities (shown in the insets of Figure 2). As can be observed, the DFSIW re-entrant cavity has an analogous but folded electric field distribution to that of the unfolded re-entrant cavity. This means that it can obtain the same resonant frequency as the unfolded re-entrant cavity while using only one quarter of the footprints [30]. Consequently, it can be concluded that the proposed DFSIW re-entrant cavity is very compact in size and hence can be applied to highly integrated systems.

### 2.3. Parameter Analysis

**Figure 3:**
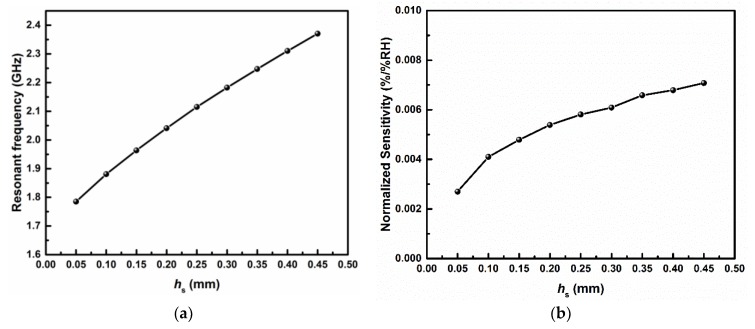
Simulated (**a**) resonant frequency and (**b**) normalized sensitivity of the sensor with varying heights of the sensitive region, *h*_s_.

In order to further investigate the influence of the geometrical parameter of the sensitive region on the performance of the sensor, the resonant frequency and normalized sensitivity of the sensor with different heights of the sensitive region *h*_s_ were simulated, and the corresponding results are shown in Figure 3a,b, respectively. The normalized sensitivity was computed using the following formulation:

(6) $$S_{n}=\left|\frac{\left[f_{r}\left(RH_{1}\right)-f_{r}\left(RH_{2}\right)\right]/f_{r}\left(RH_{1}\right)}{RH_{1}-RH_{2}}\right|,$$

where $RH_{1}$ and $RH_{2}$ represent two different RH level (in %), and $f_{r}\left(RH_{1}\right)$ and $f_{r}\left(RH_{2}\right)$ are the corresponding resonant frequencies for these two RH conditions, respectively. It is apparent from Figure 3a that the resonant frequency of the sensor was monotonically reduced with the decrease of *h*_s_. This suggests that the size miniaturization of the sensor can be further enhanced by setting a smaller *h*_s_. However, the decreasing *h*_s_ also contributed to the deterioration of the normalized sensitivity of the sensor (as shown in Figure 3b). This is due to the fact that the volume ratio of the sensitive region (or air sample) to the cavity is lowered and only limited perturbation to the original electric field can be obtained [35]. Therefore, through choosing a different value of *h*_s_, the designed sensor can be flexibly adjusted to have a more compact size or a higher sensitivity. This choice can be made according to the practical requirements. In the presented paper, the value of *h*_s_ was set to be 0.3 mm, so as to balance the relative size and sensing performance of the sensor.

## 3. Fabrication, Measurement, and Discussion

### 3.1. Sensor Fabrication and Measurement Setup

**Figure 4:**
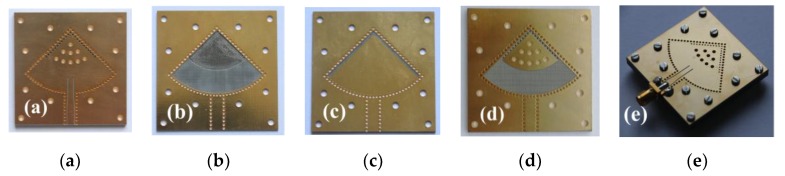
The photographs of the fabricated sensor prototype: (**a**) first layer, (**b**) second layer, (**c**) third layer, (**d**) forth layer, and (**e**) the final assembled sensor.

To experimentally demonstrate its sensing performance, the proposed DFSIW re-entrant cavity humidity sensor was fabricated using standard printed circuit board (PCB) technology. The four layers (shown in Figure 1c) were separately manufactured in the fabrication process. Both the capacitive posts on substrates 1 and 4 were built using a copper plate together with a metallized vias array embedded in the substrate. The sensitive regions were created by etching two identical circular sector grooves on the top surface of substrate 2 and the bottom surface of substrate 3. Each layer was drilled with twelve screw holes so as to align and tighten all the layers together. Finally, an SMA (sub-miniature version A) connector was soldered to the sensor for measurement purposes. The photographs of the fabricated sensor prototype, including the top views of each layer and the finally assembled sensor, are presented in Figure 4.

**Figure 5:**
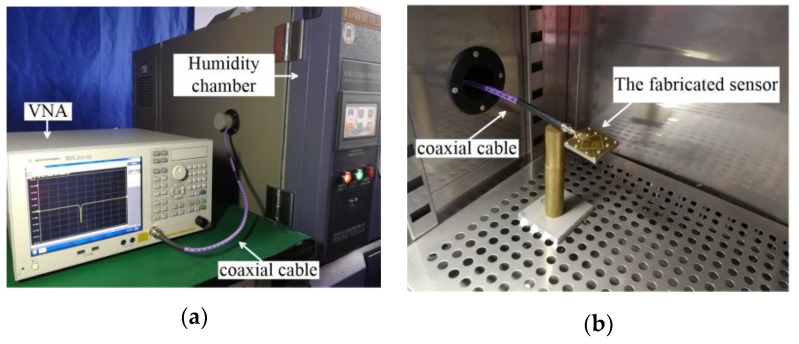
Photographs of the measurement setup for the designed DFSIW re-entrant cavity humidity sensor: (**a**) full measurement setup; (**b**) setup inside the humidity chamber.

Figure 5 depicts the experimental setup for the fabricated humidity sensor. The sensor was placed inside a humidity chamber (KW-THD-208X, KOWINTEST EQUIPMENT, Dongguan, China), where the RH level was varied from 30% to 80% with a step size of 10%. To extract the required resonant frequency, the reflection coefficient S_11_ of the sensor was measured using a vector network analyzer (VNA) (E5071C, Agilent, Beijing, China). Before the measurement, the VNA was calibrated using a standard calibration kit 85052D. In order to verify the repeatability of the sensor, the measurement for each RH condition was repeated at least four times, and the final results are the averaged values of multiple measurements.

### 3.2. Result and Discussion

**Figure 6:**
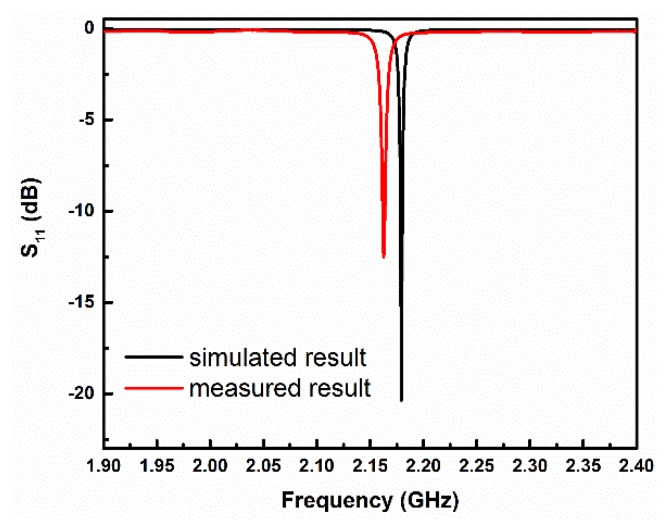
Simulated and measured reflection coefficients S_11_ of the designed humidity sensor at 30% relative humidity (RH).

Figure 6 shows the simulated and measured S_11_ of the designed humidity sensor at 30% RH. It can be seen that the measured results are in good agreement with the simulated ones except for a tiny frequency deviation and a minor decrease in return loss. The frequency deviation could be mainly attributed to the imprecise dielectric constant of the actual substrate or the fabrication tolerance (especially the tolerance on the height of the sensitive region *h*_s_). The amplitude decrease may be caused by the losses incurred from the substrate or the mismatch between the SMA connector and the cavity. The measured loaded Q-factor of the sensor was calculated to be 266. Such a high Q-factor, corresponding to a sharp resonant peak, makes it easier to trace the resonant frequency shift for small variations of RH and, therefore, improves the resolution of the sensor.

**Figure 7:**
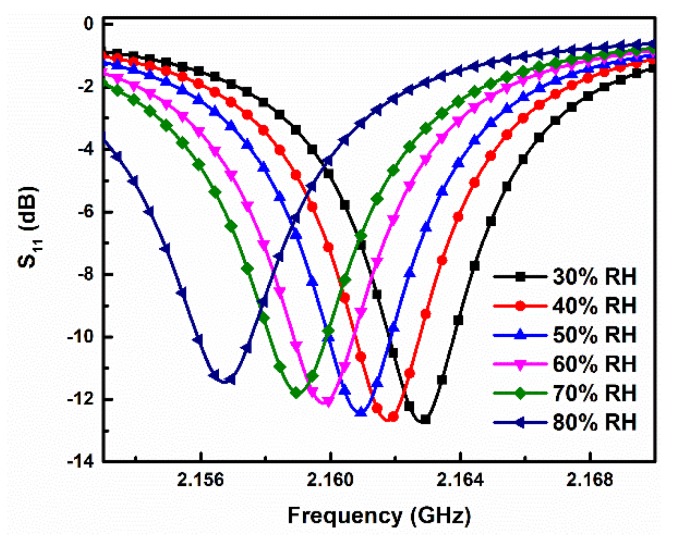
Measured reflection coefficients S_11_ of the designed humidity sensor at different RH levels.

**Figure 8:**
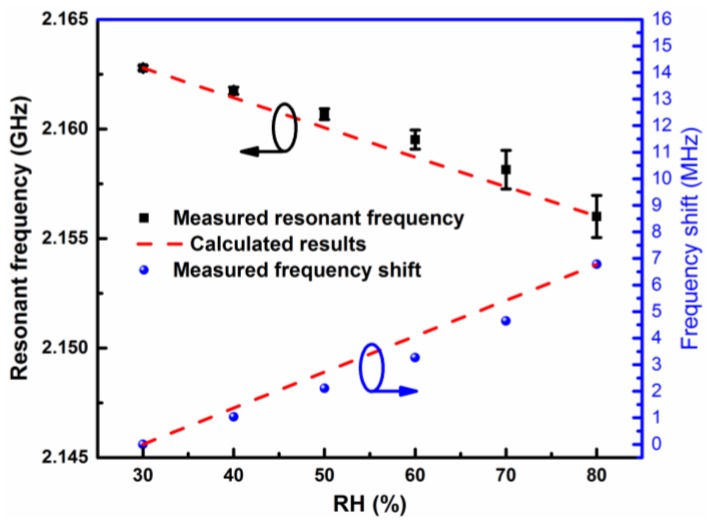
Measured resonant frequency of the designed sensor as a function of RH level.

Figure 7 shows the measured S_11_ of the designed humidity sensor at different RH conditions. It is clear that the measured resonant frequency of the sensor exhibits a continuous redshift with the increase of RH. This phenomenon is in accordance with the sensing mechanism analyzed in Section 2.1. Namely, the relative permittivity of ambient air would become higher with the increase of RH, resulting in a stronger perturbation to the induced electric field of the sensor and finally generating a larger frequency shift. Figure 8 plots the measured resonant frequency of the sensor as a function of the RH level, together with the calculated results using the theoretical model. The error bars denote the standard deviation of resonant frequency for the multiple, repeated measurements under each RH condition. As can be seen from Figure 8, when the RH level rises from 30% to 80%, the measured resonant frequency of the sensor is monotonically reduced from 2.16279 GHz to 2.15601 GHz, corresponding to an absolute frequency shift of 6.78 MHz. By using the calculation formula of$\;S_{a}=\frac{\Delta f}{\Delta \%RH}$ [22,24], the measured absolute sensitivity of our designed sensor was calculated to be 135.6 kHz/%RH. If we set the frequency step size of the VNA as 12.5 kHz (e.g., frequency range from 2.15 to 2.17 GHz with 1601 points), the resolution of the sensor is then given as 0.09% RH.

Table 2 summarizes the comparisons of some key figure-of-merit between our proposed sensor and other recently reported passive microwave humidity sensors. Considering that the operating frequencies of these sensors are different from each other, the normalized sensitivity is used here to ensure fair comparisons. Analogously, the dimension of the sensor is also given as a relative size with respect to the wavelength$\;\lambda_{0}$. As indicated in Table 2, the air-filled SIW structure in reference [23] provides the highest sensitivity but it requires a large dimension. In contrast, the QMSIW cavity in reference [24] effectively reduces the size whereas its sensitivity is too low. In comparison to these reported state-of-the-art humidity sensors, the proposed one in this paper not only has a much smaller relative size, but also offers a very competitive sensitivity (only a little lower than the air-filled SIW cavity). The measured loaded Q-factor of the proposed sensor is also at a relatively satisfactory level. Additionally, its planar SIW structure also enables it to integrate with other planar circuits easily (without any costly and sophisticated transition needed [36]). These features make the proposed sensor a potential candidate for humidity sensing applications in various fields.

## 4. Conclusions

A novel DFSIW re-entrant cavity dedicated to humidity detection is presented in this paper. The concept of incorporating folding technology with re-entrant configuration is proposed and is proven to be an effective solution for realizing ultra-compact resonator design. Compared to the conventional circular SIW cavity, the proposed structure has achieved a size miniaturization up to 85.9%. It also can be readily integrated with other planar circuits thanks to the SIW technology. The sensing mechanism of the humidity sensor was analyzed using cavity perturbation theory. It was found that the resonant frequency of the senor behaves as a function of the RH level. Consequently, the resonant frequency was employed as the signature of the sensor to monitor the RH condition of ambient air. A sensor prototype operating at 2.16 GHz was fabricated and its sensing performance was experimentally tested. The measured results demonstrate that in the RH range of 30%–80%, the designed sensor exhibits an absolute sensitivity of 135.6 kHz/%RH and a relative sensitivity of 0.00627%/%RH (normalized to the operating frequency). The proposed humidity sensor, characterized by a compact size, low cost, high Q-factor, high sensitivity, and ease of fabrication and integration, is expected to find potential applications in environment monitoring or gas sensing.

## Figures and Tables

**Table 1:** Geometrical parameters of the final optimized cavity.

Parameter	Description	Value (mm)
*L*_s_	Total length of substrate	45
*W*_s_	Total width of substrate	45
*L*_f_	Length of CPW feedline*	18.5
*W*_of_	Outer width of CPW feedline	2.77
*W*_if_	Inner width of CPW feedline	2
*r*_c_	Radius of cavity	25.2
*r*_p_	Radius of capacitive post	14.1
*d*_vp_	Diameter of via of capacitive post	1.6
*s*_vp_	Space between two adjacent vias of capacitive post	3.31
*d*_ah_	Diameter of air hole	0.5
*s*_ah_	Space between two adjacent air holes	1.25
*g*	Width of L-shaped slot	0.9
*h*_1_	Height of substrates 1 and 4	2
*h*_2_	Height of substrates 2 and 3	0.5
*h*_s_	Height of sensitive region	0.3

* CPW = coplanar waveguide.

**Table 2:** Comparison of other recently published works and this work.

Ref.	Sensor Structure	Operating Frequency (GHz)	Relative Size †	Measured Loaded Q-Factor	RH Measurement Range (%)	Normalized Sensitivity (%/%RH)
[22]-1 [22]-2	SIW cavity	4.15 3.6	(0.43$\lambda_{0}\times$0.43$\lambda_{0}$) ^§^ 0.373$\lambda_{0}\times$ 0.373$\lambda_{0}$	263.25 308	0–80	2.43$\;\times 10^{-3}$ 0.26$\;\times 10^{-3}$
[23]	Air-filled SIW cavity	7.63	0.712$\lambda_{0}\times$ 0.712$\lambda_{0}$	273.2	20–85	15.8$\;\times 10^{-3}$
[24]-1 [24]-2	QMSIW cavity^❉^	6 6.9	0.236$\lambda_{0}\times$ 0.236$\lambda_{0}$ 0.178$\lambda_{0}\times$ 0.178$\lambda_{0}$	35 86	0–80 0–70	0.608$\;\times 10^{-3}$ 0.445$\;\times 10^{-3}$
[25]	SIW interferometer	16.55	$5.4\lambda_{0}\times$ 2.54$\lambda_{0}$	2400	20–70	0.858$\;\times 10^{-3}$
This work	DFSIW re-entrant cavity	2.163	$0.161\lambda_{0}\times$ 0.161$\lambda_{0}$	266	30–80	6.27$\;\times 10^{-3}$

❉ QMSIW = quarter-mode SIW; † Only the sizes of cavity areas are calculated; § λ_0_ represents the free space wavelength at operating frequency.
